# Stray Dogs and Leishmaniasis in Urban Areas, Portugal

**DOI:** 10.3201/eid1309.070101

**Published:** 2007-09

**Authors:** Sofia Cortes, Maria Odete Afonso, Carlos Alves-Pires, Lenea Campino

**Affiliations:** * Instituto de Hygiene e Medicina Tropical, Lisbon, Portugal

**Keywords:** Leishmaniasis, stray dogs, urban areas, letter

**To the Editor**: In southern Europe, zoonotic visceral leishmaniasis caused by *Leishmania infantum* used to be considered a rural disease, but it is becoming more prevalent in urban areas. Outbreaks in urban/periurban settings are associated with the urbanization of natural zoonotic foci (*1*). The presence of a high number of stray dogs in urban/periurban settlements may contribute to the spread and increase of new infections.

A canine survey was performed twice a month from December 1, 2002, through December 31, 2003. A total of 374 dogs from urban areas of Lisbon were screened for leishmaniasis. Owners voluntarily brought 277 domestic dogs; 97 stray dogs were from public shelters. Indirect fluorescent assay was used for detection of anti-*Leishmania* antibodies using a cut-off of 1/64, and popliteal lymph node aspirates for Novy, Nicolle, and MacNeal cultures were tested (*2*).

A high overall prevalence (19.2%) of canine leishmaniasis was found, despite use of conventional tests only. The infection rate would probably have been higher had more sensitive techniques, such as molecular tools, been used. During the 1980s, Abranches et al. (*2*) performed a similar seroepidemiologic survey and found a prevalence rate of 5.5%.

Our results show an increase of canine leishmaniasis cases in Lisbon. In our study, the prevelance of infection in domestic dogs was 18.4% (51/277), and the prevelance in stray dogs was 21.6% (21/97), with no statistical difference (p = 0.48, significance level 95%). These results support the importance of the role of stray dogs in parasite transmission in Lisbon but differ from the 7.8% seroprevalence found in Madrid, where 1,803 stray dogs were studied over a 10-year period (*3*). However, the sample size and duration of both studies are different. In other urban areas of large European cities and Brazil, the existence of a high canine seroprevalence has shown an urbanization of the parasitosis (*4**,**5*). This is associated with an increase in 1-family homes with gardens in the peripheries of cities. Dogs are commonly kept in these gardens, which can provide good habitats for sandflies. On the other hand, the development of suburban areas can also lead to an increase of solid waste and deficient sanitary conditions, thus attracting infected stray dogs. The difference in percentage of domestic dogs (39.21%) and stray dogs (28.57%) that appeared healthy, although infected, was not statistically significant (p = 0.39). The percentage of apparently healthy dogs was lower than expected, as different studies have shown that more than half of the seropositive dogs are asymptomatic (*3**,**6*). Moreover, stray dogs are more likely to experience deficient health and nutritional conditions, and we thus expected larger differences between the 2 groups of animals. Of note, asymptomatic infected dogs can be a source of infection to the vectors, although symptomatic dogs are more effective reservoirs (*6*).

Along with the canine survey, from June through September a total of 488 sandflies were collected from 99 biotopes selected from the studied areas where canine or human cases have been diagnosed. The vectors were morphologically identified by standard entomologic keys (*7*) as follows: 392 (80.33%) *Phlebotomus perniciosus*, 93 (19.06%) *Ph. ariasi,* and 3 (0.61%) *Ph. sergenti*. Phlebotomine density ranged from 0.08 to 7.70 specimens/CDC trap/night. *Ph. ariasi* was found infected, reflecting an overall infection rate of 1.22% (1/82).

In Portugal, *Ph. ariasi* and *Ph. perniciosus* are the proven vectors of *L. infantum* (*8*). Although phlebotomine infection was proven in Lisbon, it was low when compared with the canine infection rate, highlighting the need for a more extensive vectorial study in these areas. From 2002 through 2006, 20 new cases of kala-azar in immunocompetent patients (16 children and 4 adults) were diagnosed in our laboratory. In spite of the number of new cases being higher in immunocompromised persons, namely, HIV-infected patients, generally only the cases of immunocompetent persons reflect natural zoonotic transmission. Immunocompromised patients can also experience the reactivation of an old latent infection or be infected by zoonotic transmission or by anthroponotic transmission without a vector. Despite some studies that have shown a direct relationship between the prevalence of leishmaniasis in canine and human populations, canine leishmaniasis is much more prevalent and more widely distributed than visceral leishmaniasis, and it does not strongly correlate with the prevalence in humans (*6*). Moreover, *Ph. ariasi* and *Ph. perniciosus* are known to be preferentially zoophilic.

In domestic dogs, if the owner takes preventive measures, the infection risk may be reduced. Stray dogs, however, are an easier target for infection and sandfly biting due to precarious physical conditions and outdoor living habits that make canine leishmaniasis control much more difficult.

In conclusion, sanitary conditions and animal health must be improved to prevent the transmission risk of leishmaniasis by this group of animals. The absence of surveillance or preventive measures and equilibrium rupture in the ecologic system could contribute to the emergence of human leishmaniasis in urban areas.
